# Impact of a person-centered intervention for patients with head and neck cancer: a qualitative exploration

**DOI:** 10.1186/s12912-018-0319-6

**Published:** 2018-11-21

**Authors:** Ingalill Koinberg, Elisabeth Hansson Olofsson, Eric Carlström, Lars-Eric Olsson

**Affiliations:** 1 The Sahlgrenska Academy – Institute of Health and Care Sciences, University of Gothenburg, Gothenburg, Sweden; 2 Centre for Person-Centred Care (GPCC), Gothenburg University, Gothenburg, Sweden; 3 Department of Oncology, Sahlgrenska University Hospital, Gothenburg, Sweden

**Keywords:** Head and neck cancer, Nursing, patient’s experience, Person-centered care, Transition theory

## Abstract

**Background:**

People affected by head and neck cancer (HNC) experience a variety of multifaceted health-related problems during the treatment process, based on both the disease and side effects, several years after the treatment is complete. This study investigated a person-centred intervention using transition theory as a framework.

**Aim:**

Thus, the aim of the present study was to explore patients’ experience of the transition and person centred care from diagnosis to the end of the treatment period.

**Methods:**

Interviews were conducted with 12 persons included in the person-centred intervention group. The patients were recruited from a randomised controlled study. We used a directed deductive content analysis as an analysis method.

**Results:**

There was a distinct transition between being a healthy person to being diagnosed with a serious disease. The majority of the participants felt that the diagnosis had put their lives in the balance; they felt both healthy and sick at the same time, and all participants described that their symptoms and side effects were the worst possible and totally unexpected. Of great importance was the health-care plan, comprising self-management goals which were formed in partnership between the patient and the nurse. The participants experienced that their interaction and engagement with lay persons and healthcare professionals supported a gradual acceptance of the situation and a sense of relief with a kind of awareness of the disease.

**Conclusion:**

The intervention played a significant role in promoting a healthy transition. Person-centredness and transition theory can help healthcare professionals to be more confident and resourceful in supporting people affected by HNC.

## Background

There are several quantitative studies that describe a variety of multifaceted health-related quality of life problems (HRQoL) for people affected by head and neck cancer, based on both the disease and the side effects of treatment. The medical treatment usually consists of both chemotherapy and radiotherapy, and the most commonly described physical problems are pain, difficulty swallowing, nausea, vomiting, fatigue, and mouth opening and eating problems during treatment, and the complications can persist for several years after the end of treatment [1–5]. Psychosocial problems are also common and are expressed in the form of anxiety and depression, reduced social contact, and long-term sick leave [2, 4, 6, 7].

Middle-range transitions theory describes the characteristics of life transitions and can be a framework that provides understanding, expectations, knowledge and ability to plan for treatment, recovery and rehabilitation [8, 9]. According to Meleis [10], a healthy transition can be identified through the involvement of new roles and focus on new forms of interaction. Skärsäter and Willman [11] note that transitional planning is a viable way to achieve goals that provide adequate care and health promotion to achieve a good quality of life for patients. Another study by Joly [12] emphasises that nurses must act as leaders to support the transition to adulthood for young people under the influence of complex medical issues.

Transition is a central concept in nursing as well as in person-centred care (PCC). Person-centred care is based on three concepts: the patient’s narrative establishes a partnership and promotes shared decision-making; the documentation of that narrative in patient records validates the partnership; and, throughout, it is acknowledged that the person and not the disease must be the focus [13]. PCC aims to interpret behaviour and symptoms from the patient’s point of view in order to individualise the care. The prerequisite for person-centred care is the telling of the patient’s story of her condition, a patient narrative that is created through the dialogue between patients and health professionals. The focus of the story’s content is the patient’s personal experiences and interpretation of their symptoms, how these symptoms affect their daily life, and the opportunities (resources) that the patient can see in their lives. The story is used as a basis for the planning of care and is written down in the form of a health-care plan. Dwamena et al. [14] concluded in a Cochrane review that interventions to promote patient-centred care within clinical consultations are effective in transferring patient-centred skills to providers across studies. The most important instrument in PCC is to take up the patients’ history about the their prerequisites, values and preferences [13, 15]. The outcome of a transaction cost study revealed that a person-centred care intervention has been successful in coping with issues relating to unannounced patients, needing fewer resources, and providing better services than the traditional ad hoc organisation [16].

There is a need for qualitative studies using a theory that reflects the difference and complexities of vulnerable patient’s response to transition. Consequently there are few studies that have investigated patients’ experiences of symptoms and their impact on HRQoL [7, 17, 18] nor are there any describing effective interventions to improve HRQoL in HNC patients. Semple et al. [17] concluded in a Cochrane review including seven trials with a total of 542 participants that there is insufficient evidence to support the use of psychosocial interventions for HNC patients. Cancer care teams who have a nurse as their coordinator are faced with the challenge to provide person-centred care by involving the patient in the decision-making and management of their condition by taking the patient’s history and designing a health-care plan [19]. There is, to our knowledge, no qualitative study describing HNC patients’ transition through, and their experiences of, the care that they receive during the diagnosis and treatment period. Thus, the aim of the present study was to explore patients’ experience of transition and person centred care from diagnosis to the end of the treatment period.

## Methods

### Design

The present study used a descriptive qualitative design with interviews as a data collection method, and directed deductive content analysis as the analysis method [20]. The twelve interviews were conducted in autumn 2014 until spring 2015.

### Participants and settings

Twelve patients were approached by means of a letter of enquiry and verbally by the nurses at the outpatient clinic and these individuals were in different phases of their treatment process. All of the patients who were invited to take part agreed to participate. The patients were diagnosed with various kinds of head and neck cancer, such as tonsil cancer (*n* = 8), oropharyngeal cancer (*n* = 2), nasopharynx cancer (*n* = 1), and tumor collie (*n* = 1). The participants were treated with surgery combined with radiation and chemotherapy. Ten patients were diagnosed with an infection by human papillomavirus (HPV) and were diagnosed with an HPV-positive HNC cancer. For two patients, a blood sample was not taken, and it was unclear whether the patients were HPV-positive. There were seven women and five men included in the study, and their ages ranged between 49 and 64 years with an average age of 57 years. Ten of the patients were employed, and two were retired. The interviews were conducted after the treatment was completed, between approximately six and 12 months after diagnosis.

This study was a part of a randomised controlled trial (RCT) where patients were recruited from an oncologic department specialising in head and neck cancer at a University hospital in Sweden [21]. The twelve interviewed patients were all recruited from the intervention group, all respondents agreed to participate. Gothenburg Person Centred Care (gPCC) intervention consisted of a high continuity and accessibility to one of two specialised nurses. The nurse specialists recorded each patient’s history and together they developed a health plan. They coordinated the care, gave advice and support about symptoms and side effects, and about how to resolve eating problems during treatment. The nurses provided both the patients and their relatives with information and support when needed.

A typical treatment with chemo-radiotherapy consists of pre-treatment preparation with dental care, nutritional advising, blood tests, fixation and scanning for radiotherapy dose-planning. The primary tumour and affected lymph nodes are to be irradiated to a higher dose and a prophylactic dose level will be used for lymph nodes bilaterally. The dose is divided into 34 fractions in six weeks. A weekly dose of chemotherapy is given intravenously for patients in good general health with locally advanced tumours. Typical side effects of this treatment will be pronounced mucositis after approximately three weeks, with pain, difficulties with oral food intake, loss of taste, dry mouth, and risk of weight loss. The chemotherapy treatment could cause nausea and vomiting and an increased risk of serious infections. The treatment often starts on an out-patient basis, but approximately half of the patients must remain in hospital for more intensive care and nutritional support after some weeks.

### Interviews

To acquire deeper knowledge of the patients’ experiences of the impact of the care they received, semi-structured interviews were conducted covering the following areas: “Can you tell me about your experience of care from the time you sought medical care until you got your diagnosis and treatment?” “Can you tell me about how the diagnosis and treatment has affected you psychologically, socially, emotionally, and physically?” “Can you tell me about the positive and negative events associated with health care?” One researcher performed the interviews (ILK). All participants were informed, both verbally and by being given written information, and signed a consent form. The researcher created an atmosphere of trust and emphasised that each person’s contributions were important. Participants were encouraged to speak freely and in their own terms. The researcher is familiar with oncology and oncology care and has, in the analysis, endeavored to avoid the incorrect interpretation of data. The interviews took place in a conference room in the oncological clinic and each lasted for approximately one hour. The interviews were tape-recorded and transcribed verbatim. Data from the interviews were rich and, as a result, the material was separated into two parts: one relating to the period before the diagnosis and the treatment period; and the other relating to the period after treatment, including recovery and rehabilitation.

### Analysis

Directed deductive content analysis [20] A coding framework was developed, which represented the Nature of transition (properties), Transition conditions (personal conditions) and Patterns of response (process indicators) (Table 1). In the next step, the research question guided the identification of meaning units within the textual data; then, two of the authors (IK and LEO) discussed the meaning units in relation to transitions theory by means of critically analysing, questioning, and comparing the statements in order to achieve trustworthiness [20]. The two authors confirmed the analysis by conducting a random control process on a selection of the transcribed texts [20]. The analysis process described by Hsieh and Shannon [20] was then followed. In the first step, we prepared the data and the main question from the interviews was selected. In the next step, we defined the coding units into individual themes and developed categories and a coding scheme. Thereafter, we tested the coding scheme on a sample of text. In the last step, we rechecked the consistency of the coding and conclusions were drawn from the data. Data that could not be coded by the transitions theory components were identified and analysed later, to determine whether they represented a new category or a subcategory of an existing code. The purpose of this study was to explore describe the transition from the time before diagnosis to the end of the treatment period. This emerged in the analysis as the following categories: awareness, engagement, change and difference, and critical points and events. Personal conditions included preparation and knowledge, together with such process indicators as developing confidence and coping and interacting.

**Table 1:** Transitions theory (modified from Meleis’ transitions theory): essential properties of transition experiences

Properties	
Awareness:	Awareness was related to a sense or feeling of illness and a gradual preparedness and understanding of the disease and how the person perceived their situation.
Engagement:	Engagement is defined as the degree to which a person demonstrates commitment to the process from diagnosis to treatment period and how they perceive the transition from being healthy to being sick.
Change and difference:	Change and difference relates to the participants’ perceptions of the changes they experienced during the period between diagnosis and treatment.
Critical points and events:	Transitions experiences involved critical turning points or events. Critical points were often associated with increasing awareness of change or difference, or more active engagement in dealing with the transition experience. In addition, there were final critical points, which were characterised by a sense of stabilisation in new routines, skills, lifestyles, and self-care activities.
Personal conditions	
Preparation and Knowledge:	Being prepared and having different life experiences facilitates the transition, while the lack of preparation can inhibit the process. Interconnected with preparation is the knowledge of what to expect during the transition and what strategies can be helpful for dealing with it. Knowledge about the possibility of getting well affect positively.
Process Indicators	
Developing confidence and Coping:	Reflects the nature of the transition process to which there is a pattern that indicates that people experience a greater degree of trust and understanding towards medical care and treatment. Persons use their resources and capacity to develop strategies to deal with their situation and make it easier to live with limitations.
Interacting	Interaction and confirmation from the medical staff and next of kin have been clarified through interaction and reflection on new and emerging relationships and was necessary to achieve a successful transition.

### Ethical considerations

All participants received a letter describing the purpose and procedure of the study. The letter also stated that their participation was voluntary, that the study records would be kept confidential, and that their contributions would be unidentifiable in the final report. The study was explained and patients were asked by the physician to participate after a consultation meeting. All participants then signed a consent form. The Regional Ethics Committee in Gothenburg approved the study (Dnr: 025–12).

### Findings

The findings are reported under the transitions theory headings, based on Meleis’ framework, and each heading is supported by subthemes; some of which are illuminated by quotations from the participants (see Table 1). Of the transition properties outlined by Meleis, awareness, engagement, change and difference, and critical points and events emerged in our findings. The personal conditions that arose included preparation and knowledge, and the process indicators that the participants used included developing confidence and coping and interacting with healthcare professionals and family members.

### Awareness and engagement

The concepts of awareness and engagement were closely linked together and seemed crucial in order for the patients to understand what had happened. Awareness was related to perception, knowledge, and recognition of the transition experience. There was a distinct transition in going from being a healthy person to being diagnosed with a serious disease. Engagement was defined as the degree to which a person demonstrates commitment to the process from diagnosis to the treatment period, and how they perceive the transition from being healthy to being sick. Most patients already had a feeling that something was wrong before being diagnosed, and they described how they went to the Primary Care Center on several occasions before their symptoms were taken seriously. “*I probably suspected this myself, I did. I would have done it for a long time that this cannot be right, so for me it was probably just a confirmation*”. It was essential that they were taken seriously and that their needs were properly addressed. As one of the patients described: “*I want to know what happens to me, I want to know everything. So that way, so, I’m involved, but at the same time, I am completely vulnerable anyway*”*.* Several participants felt that they were given time for questions and that they were prepared for treatment. However, other participants expressed that it went too quickly and that there was no time for reflection; they felt uncertain when the information they were given was too general: “*It went fast after you tried the stuff and then I came in here and got my diagnosis*”.

In this study, informants expressed the problems they had experienced clearly, and some individuals were in a very vulnerable situation but felt that the support from the nurses was very helpful in the transition process. They pointed out that the first meeting with the nurse was important; the nurse had listened carefully and explained why the treatment was necessary and what symptoms and side effects could be expected: “*Nurses helped to put together the different threads into something more understandable to others, so everyone said, everyone said exactly what they said was really important but you could not get all these threads together, solve the threads here and there*”. The informants had many sources of information, such as relatives, close friends, and the internet, but they also expressed a need for contact with others in a similar situation. “*Then I met people who were just the same, at my age so I talked to them every day when I got a chemotherapy*”*.* The majority of the participants experienced that the engagement from lay persons and healthcare professionals supported a gradual acceptance of the situation and a sense of relief, along with a kind of awareness of the disease.

### Change and difference

Changes were related to critical events or to disruption in routines, relationships, or perceptions. The data revealed that all participants experienced a change in their perceptions of their future and their situation, and this also had an impact on their social life. All participants experienced the medical treatment with chemotherapy and radiation therapy as being very tough and stressful, and the majority of participants expressed how ill they felt during treatment and that it took much effort to concentrate on trying to keep their heads above water. All participants described that their symptoms and side effects were the worst possible and that they were totally unexpected. They described the tremendous fatigue that affected them during the treatment phase, how it was difficult to climb the stairs to eat food, and spend time with their families, and how they slept up to 18 h per day. One respondent had goals and targets marked out for what he would do when the treatment was completed and he had planned a number of activities that he would do when he was healthy: “*have a lot of new goals this I do for the summer I want to see light at the end of the tunnel*”. How serious the participant perceived their condition to be was linked to their past experiences, such as how they dealt with the situation, how it affected their social life, working life, and bodily changes. Related to the complex treatment, the participants understood that they had many difficult decisions to make, either alone or together with their families, and most of them felt lucky to have someone to share such decisions with. The nurses became an important partner in the treatment process in order to understand how the treatment affects the vital organs and how it manifests itself in symptoms and side effects: “*someone has said that the treatment will take X number of months and contain X number of radiation and X number of chemotherapy, it is still extremely abstract because is something that you have never ever done before*”.

### Critical points and events

Getting the diagnosis was perceived as a critical point and as one of life’s most dramatic events. Some participants were shocked and could not assimilate any further information. A majority of the participants felt that the diagnosis had put their lives in the balance, in that they felt both healthy and sick at the same time. “*It was a bit confusing, when it became clear that you had cancer, you were also told that you should recover even if it is a tough treatment*”. The interaction and the assurance from the physician and nurses was felt to be necessary to obtain further information and to become aware of what the diagnosis meant and to be prepared for future treatment: “*The announcement that I was going to accomplish this was important for me*”. For most of the participants, it was important that such difficult conversations took place in an appropriate way that there was time for questions, and that the healthcare professionals were present, empathetic, and dedicated in this difficult situation: “*I sought support by looking for sympathetic faces and I was lucky to get one of the doctors whom I perceived as sympathetic*”*.* Another critical point was the side effects after radiotherapy and chemotherapy, especially the problems with eating that appeared after the treatments ended. All participants received some form of radiation injury in the neck region, which in turn resulted in pain: “*Then it hurt like the devil in the mouth, that was so difficult and I did not think any of these pain reliefs helped*”*.* Chemotherapy had the side effect of nausea and vomiting, which in turn made it next to impossible to eat normal food or to eat food quickly.

### Preparation and knowledge is the prerequisite for developing confidence and coping strategies

Of great importance was the health-care plan, comprising self-management goals which were formed in partnership between the patient and the nurse. Each patient was encouraged to reflect on the self-management goals, how to reach them, and to anticipate barriers: “*Although the treatment was considered to be the toughest, I was judged to be able to handle it and recover, which was very safe*”. Participants described how old strategies were applied and that new strategies were developed and how they used this knowledge to cope with the situation: “*I think it will be alright, says my doctor and my nurses despite my cancer, and I will be fine and everything will be fine, I’m going to be treated*”. Several participants used their resources and capacity to develop strategies to deal with their situation and make it easier to live with their functional limitations. An important factor to take into account was to ensure that the symptoms and side effects of treatment did not compromise the perceived balance between life activities: “*One stroke. I was punished mentally and the body was like a tatter and I could not hurry. You saw how the muscles just disappeared on the body and then you thought that, how are you going to do this after that? Either I have taken 10 years of life or I got 10 years extra*”. Although the prognosis for patients diagnosed with HPV-positive HNC are good and the possibility of cure is high, several participants worry about whether they belong to the percentage who do not survive: “*It was a lot of tough trips I really felt both physically and mentally. I went home anyway, and then I was hysterical when I got home, I did not want to go home because I did not think I could handle it*”.

### Interaction

Strategies were clarified through interaction and reflection in the conversation with the nurse and enabled new and emerging relationship for both parties. The participants felt that the treatment was extremely abstract, with a number of X-rays and chemotherapy sessions which had to be explained by the nurses, who also started to become a sort of therapist: “*I was able to speak freely and because they are nurses, they could explain in an understandable way and I felt well taken care of*”.

How to manage symptoms, side-effects, and daily life problems was discussed during every session with the specialist nurse. If the participant wanted, and if it was possible, options and modifications were discussed, as well as how to manage intensity during the contacts, either by telephone or in personal meetings: “*I thought it was nice when the nurse called and heard how I was doing*”. Participants felt well cared for by the nurses, who they could call whenever they wanted on a direct telephone number if they had any questions about anything concerning their treatment and wellbeing. They were grateful for the opportunity to call at any time and they felt that the nurses really cared about them. The participants stressed the importance of being recognised and that there was someone who listened to them when they called and to reassure them that it would get better. Several participants described that they received solid information from the physician that was linked to a positive vision of the future. A sense of mutual confidence was described when the interaction between the physician and patient were developed: “*I received good care both by doctors and nurses, I understood that they take good care of us patients and not only be seen as a social security number*”. Family members and friends were essential in order to manage daily life problems and to deal with anxiety and depressive feelings: “*I just felt worse and worse and I almost felt panic and then it felt incredibly important to have someone who I could talk to*”. Several respondents described the great support they had in their husbands, wives or friends:

“*His support was enough when I got home and we got to talk through everything after it was clear and we have very open and talked all the time very openly about this*”.

### Methods discussion and limitations

While this is one of few qualitative studies using Meleis’ (2010) transitions theory framework, our findings need to be interpreted in light of its limitations. Skärsäter and Willman [11] also confirmed that the transitional framework was useful in psychiatric care, as they described the transition process as being moveable, back and forth, which is confirmed in their study and among the participants in this study through the participants’ statements. Transitions theory can be seen as a validation and allows for an exhaustive description of the healthcare process and phenomenon. Trustworthiness and credibility has been achieved by the researchers systematically reflected and worked together in conjunction during the knowledge construction at each step of the research process. We were aware that the researchers’ individual background and position could affect both the interviews and the analysis. The content analysis method was considered appropriate for this purpose, as shown in the results, which we consider to be clinically relevant [22].

Both the interviewer, who is the first author (ILK), and the researcher (LEO) were aware of the risk of researcher’s bias and took care in the analysis and interpretation to minimise this risk. In addition, every effort was made to ensure that the results and conclusions of this study were supported by the data and shaped by the participants rather than the researchers’ bias, motivations or interests. Finally, as this study was part of an RCT, it is possible that the interview questions used were affected by a deep desire to learn more about the patients’ experiences of care. This may be a limitation in this current analysis, as might be the small number of participants included in this study.

## Discussion

The findings indicate that the patients were treated as individuals and that they did experience person-centred care. In addition, it can be inferred that the interaction between nurses and physicians during the treatment process played an influential role in the extent to which the patients experienced a healthy transition. The transition process was described from each person’s emerging awareness about his/her situation, which occurred in connection with the diagnosis and continued throughout the treatment process. Their level of engagement was largely influenced by the extent to which they participated in the processes involved in the treatment period. Interaction with family and caregivers allowed the patient to manage change and difference, and critical points during the transition period. Being prepared and having sufficient knowledge was essential for understanding what to expect during the transition and what strategies they can adopt to help them to cope with it. All concepts were closely intertwined, but the interaction with nurses and doctors was necessary and affected the transition and enabled the patient to develop self-confidence and management strategies.

The findings suggest that awareness may involve an inner understanding, eventually reaching a so-called rational consciousness or a sudden deeper understanding that we had not previously understood or been aware of [9]. To be aware can also be based on personal experience or the notion that we have a sense of something and/or have previously trained our minds to attune to this awareness.

The diagnosis represents a stigma in itself, because head and neck cancer is usually associated with an unhealthy lifestyle, such as tobacco or alcohol use, and is usually accompanied by a poor prognosis. The majority of patients in the present study had been diagnosed with HPV-positive HNC, which means that they had a better clinical outcome than patients diagnosed with HPV-negative HNC [23]. This creates a confusing situation for the patient, not only because they have been diagnosed with cancer, but also because they have been promised a good prognosis, which makes them feel both sick and healthy at the same time. In line with Moore, Ford and Farah [24], this awareness increases gradually, from the discovery of cancer, to the emotional reactions, to the treatment and its side effects.

Several participants in our study expressed that awareness was linked to a commitment that was based on personal experience and feelings. The level of awareness influenced the levels of engagement, and engagement cannot occur without awareness.

The participants in the study expressed that the personal engagement from the healthcare professionals helped them to practise self-care to a greater extent. The nurses and physicians managed to convince the patients that they were capable persons with resources, which contributed greatly to the coping strategies they developed while undergoing treatment. Therefore, it is important to prepare patients for the treatment period and for the period after treatment by giving them adequate information at the right time, in the right place, and by professionals on an individual basis [25–27].

All transitions involve change, whereas not all change is related to transition. All participants described the impact of the diagnosis as being an abrupt change in their lives. The majority of the participants expressed how bad they felt during treatment, and that it took much effort to concentrate on trying to keep their heads above water. All participants described that their symptoms and side effects were the worst possible and that they were totally unexpected. The results of this qualitative study support the outcomes from the RCT study in that the worst health-related problems were noted four weeks after treatment started [21].

However, the transition was a long-term process which gradually customised the individuals for new roles and situations. Changes and differences related to their approach to the diagnosis and the deeper meaning they developed in having been diagnosed with cancer. The experience of having been diagnosed with a serious illness and its effects on daily and social life but with the possibility of recovering contributed to the fact that most people looked forward to a relatively bright future. It may be useful for nurses to consider a person’s level of comfort and mastery in dealing with such change and difference [9].

Critical points were frequently associated with an increased awareness of change, and the experience was generated by the participants’ active involvement in managing the transition. Getting a cancer diagnosis was experienced as a shock to most participants and making a deal between life and death and survival seems to be the strongest coping strategy [28]. Patients feel vulnerable throughout the care process because the transformation affects life, health, and relationships [9].

Moreover, there was the final critical point, which is characterised by a stabilization of the new procedures, skills, lifestyles, and self-care activities. Some participants felt that the critical events were associated with an increased awareness of change. Their existence stabilised with setting new routines, a change in lifestyle, and in finding ways to cope with the situation.

Over the past three decades there has been an increased incidence of the number of cases of HNC, which can be explained by an increase in HPV-positive (human papilloma virus) infection [28]. Patients diagnosed with HPV-positive HNC cancer have a better clinical outcome than patients diagnosed with HPV-negative HNC and other head and neck cancer patients, which raises the question about the chemo-radiotherapy given to all head and neck cancer patients, suggesting that less intensive treatment could reduce the serious side effects and yet maintain survival [23, 29, 30].

Being prepared and having different life experiences and knowledge of what to expect and what strategies to adopt can help to manage the situation and positively affect the outcomes. Personal conditions, such as different life experiences, facilitated the transition. The nurses managed to convince the patients that they were capable persons with resources, which contributed much to the coping strategies that the participants developed while undergoing the treatment, and both physicians and nurses provided a basis and created a trustful relationship by giving the participants all the time they needed for questions. The person’s ability to develop confidence in the healthcare professionals depended on their own individual qualities, and it was found that this confidence depends explicitly or implicitly on the other’s trustworthiness. When evaluating the health-care plan, the severity of symptoms and side effects was discussed. The updating of the health-care plan was a continuous process, resulting in the experience of a higher degree of trust and understanding about medical care and treatment for the participants.

All participants used their own individual ability, resources and capacity to develop strategies to deal with their situation and make it manageable. Confidence increased progressively over time, and reflected the nature of the transition process. There was a pattern that indicated that the participants experienced a greater degree of trust and understanding towards healthcare professionals in relation to medical care and treatment. A further effect that we observed is that the participants’ perceptions of the quality of care and their sense of security increased, which is often possible when patients experience the care as person-centred, and this is strongly associated with patient ratings of care [31].

Interaction and confirmation from the medical staff and next of kin have been clarified through interaction and reflection on new and emerging relationships and was necessary to achieve a successful transition. The trajectory of care for head and neck cancer patients is a demanding process characterised by a series of ups and downs, and to meet these patients’ needs, the care must provide greater consistency and continuity [32]. Involving and supporting the patient’s family is part of the recovery process, and several participants in our study expressed that this stressful experience can be relieved by talking with other people in the same situation.

Through interaction, the meaning of the transition and behaviours developed during the process was clarified and confirmed. The nurses discussed goals, strategies, resources, capacity, and barriers in order to suit each person’s own circumstances as a part of the health-care plan. Each patient was encouraged to reflect on the self-management goals, how to reach them, and to anticipate barriers.

## Conclusion

Person-centeredness and transition theory can help healthcare professionals to be more confident and resourceful in supporting patients affected by head and neck cancer.

### Nursing implications

By giving the patient the opportunity to present himself as a person and that the presentation takes place in the form of a story establishes a common starting point. Shared decision-making encourages and allows patients to take an active part in finding solutions to their problems and identify their resources. A person-centred approach increases the likelihood of a coherent chain of care where patients themselves do not have to navigate their way through the multi-professional organization of hospitals and other healthcare institutions.

## Abbreviations

gPCC: Gothenburg Person Centered Care
HNC: Head and Neck Cancer
HPV: Human Papilloma Virus
HRQoL: Health related Quality of Life
PCC: Person Centered Care
RCT: Randomised Cotrolled Trial

## References

[1] Molassiotis A, Rogers M (2012). Symptom experience and regaining normality in the first year following a diagnosis of head and neck cancer: a qualitative longitudinal study. Palliat Support Care.

[2] Neilson K, Pollard A, Boonzaier A, Corry J, Castle D, Smith D (2013). A longitudinal study of distress (depression and anxiety) up to 18 months after radiotherapy for head and neck cancer. Psychooncology.

[3] Oksam IM, Verdonck-de-Leeuw IM, Aaronson NK, Witte BI, de Bree R, Doornaert P (2013). Prospective evaluation of health-related quality of life in long-term oral and oropharyngeal cancer survivors and the perceived need for supportive care. Oral Oncol.

[4] van der Meulen IC, May AM, de Leeuw JR, Koole R, Oosterom M, Hordijk GJ, Ros WJ (2014). Long-term effect of a nurse-led psychosocial intervention on health-related quality of life in patients with head and neck cancer: a randomised controlled trial. Br J Cancer.

[5] Wells M, Donnan PT, Sharp L, Ackland C, Fletcher J, Dewar JA (2008). A study to evaluate nurse-led on-treatment review for patients undergoing radiotherapy for head and neck cancer. J Clin Nurs.

[6] Murphy BA, Ridner S, Wells N, Dietrich M (2007). Quality of life research in head and neck cancer: a review of the current state of the science. Crit Rev Oncol Hematol.

[7] Semple CJ, Dunwoody L, Kernohan WG, McCaughan E, Sullivan K (2008). Changes and challenges to patients’ lifestyle patterns following treatment for head and neck cancer. J Adv Nurs.

[8] Meleis AI (2010). Transitions theory: middle-range and situation-specific theories in nursing research and practice.

[9] Meleis AI, Sawyer LM, Im EO, Hilfinger Messias DK, Schumacher K (2000). Experiencing transitions: an emerging middle-range theory. Adv Nurs Sci.

[10] Meleis AI (2016). The undeaning transition: toward becoming a former dean. Nurs Outlook.

[11] Skärsäter I, Willman A (2006). The recovery process in major depression: an analysis employing Meleis’ Transition Framework for deeper understanding as a foundation for nursing interventions. Advan Nurs Sci.

[12] Joly E (2016). Integrating transition theory and bioecological theory: a theoretical perspective for nurses supporting the transition to adulthood for young people with medical complexity. J Avan Nurs.

[13] Ekman I, Wolf A, Olsson LE, Taft C, Dudas K, Schaufelberger M, Swedberg K (2012). Effects of person-centred care in patients with chronic heart failure: the PCC-HF study. Eur Heart J.

[14] Dwamena F, Holmes-Rovner M, Gaulden CM, Jorgenson S, Sadigh G, Sikorskii A, Lewin S (2012). Interventions for providers to promote a patient-centred approach in clinical consultations. Cochrane Database Syst Rev.

[15] Olsson LE, Jakobsson Ung E, Swedberg K, Ekman I (2013). Efficacy of person-centred care as an intervention in controlled trials – a systematic review. J Clin Nurs.

[16] Carlström ED, Hansson Olofsson E, Olsson L-E, Nyman J, Koinberg I-L (2015). The unannounced patient in the corridor: trust, friction and person-centered care. Int J Health Plann Manag.

[17] Semple C, Parahoo K, Norman A, McCaughan E, Humprhis G, Mills M (2013). Psychosocial interventions for patients with head and neck cancer (review). Cochrane Database Syst Rev.

[18] de Leeuw J, Larsson M (2013). Nurse-led follow-up care for cancer patients: what is known and what is needed. Support Care Cancer.

[19] Ulin K, Olsson LE, Wolf A, Ekman I (2016). Person-centred care – an approach that improves the discharge process. Eur J Cardiovasc Nurs.

[20] Hsieh HF, Shannon SE (2005). Three approaches to qualitative content analysis. Qual Health Res.

[21] Hansson-Olofsson E, Carlstrom E, Olsson L-E, Nyman J, Koinberg I (2017). Can a person-centred-care intervention improve health-related quality of life in patients with head and neck cancer? A randomized, controlled study. BMC Nurs.

[22] Malterud K (2006). Theory and interpretation in qualitative studies from general practice: why and how?. Scand J Public Health.

[23] Dalianis T (2014). Human papillomavirus (HPV) and oropharyngeal squamous cell carcinoma. Presse Med.

[24] Moore KA, Ford PJ, Farah CS (2014). “I have quality of life… but…”: exploring support needs important to quality of life in head and neck cancer. Head Neck.

[25] Llewellyn CD, McGurk M, Weinman J (2005). Are psycho-social and behavioural factors related to health related-quality of life in patients with head and neck cancer? A systematic review. Oral Oncol.

[26] Pollock K, Cox K, Howard P, Wilson E, Moghaddam N (2008). Service user experiences of information delivery after a diagnosis of cancer: a qualitative study. Support Care Cancer.

[27] Jabbour J, Milross C, Sundaresan P, Ebrahimi A, Shepherd HL, Dhillon H (2017). Education and support needs in patients with head and neck cancer: a multi-institutional study. Cancer.

[28] Reich M, Licitra L, Vermorken JB, Bernier J, Parmar S, Golusinski W (2016). Best practice guidelines in the psychosocial management of HPV-related head and neck cancer: recommendations from the European head and neck Cancer Society’s make sense campaign. Ann Oncol.

[29] Goodman MT, Saraiya M, Thompson TD, Steinau M, Hernandez BY, Lynch CF, Unger ER (2015). Human papillomavirus genotype and oropharynx cancer survival in the United States of America. Eur J Cancer.

[30] Ramqvist T, Grun N, Dalianis T (2015). Human papillomavirus and tonsillar and base of tongue cancer. Viruses.

[31] Edvardsson D, Watt E, Pearce F (2017). Patient experiences of caring and person-centredness are associated with perceived nursing care quality. J Advan Nurs.

[32] Larsson M, Hedelin B, Athlin A (2007). A supportive nursing care clinic: conceptions of patients with head and neck cancer. Eur J Oncol Nurs.

